# Weight Loss Outcomes Associated With Semaglutide Treatment for Patients With Overweight or Obesity

**DOI:** 10.1001/jamanetworkopen.2022.31982

**Published:** 2022-09-19

**Authors:** Wissam Ghusn, Alan De la Rosa, Daniel Sacoto, Lizeth Cifuentes, Alejandro Campos, Fauzi Feris, Maria Daniela Hurtado, Andres Acosta

**Affiliations:** 1 Precision Medicine for Obesity Program, Division of Gastroenterology and Hepatology, Department of Medicine, Mayo Clinic, Rochester, Minnesota; 2 Division of Endocrinology, Diabetes, Metabolism, and Nutrition, Department of Medicine, Mayo Clinic, Jacksonville, Florida

## Abstract

**Importance:**

No retrospective cohort study has assessed the effectiveness of semaglutide at doses used in randomized clinical trials to treat obesity (ie, 1.7 and 2.4 mg).

**Objective:**

To study weight loss outcomes associated with semaglutide treatment at doses used in randomized clinical trials for patients with overweight or obesity.

**Design, Setting, and Participants:**

This cohort study, conducted at a referral center for weight management, retrospectively collected data on the use of semaglutide for adults with overweight or obesity between January 1, 2021, and March 15, 2022, with a follow-up of up to 6 months. A total of 408 patients with a body mass index (BMI) of 27 or more were prescribed weekly semaglutide subcutaneous injections for 3 months or more. Patients with a history of bariatric procedures, taking other antiobesity medications, and with an active malignant neoplasm were excluded.

**Exposures:**

Weekly 1.7-mg or 2.4-mg semaglutide subcutaneous injections for 3 to 6 months.

**Main Outcomes and Measures:**

The primary end point was the percentage of weight loss. Secondary end points were the proportion of patients achieving weight loss of 5% or more, 10% or more, 15% or more, and 20% or more after 3 and 6 months and the percentage of weight loss for patients with or without type 2 diabetes after 3 and 6 months.

**Results:**

The study included 175 patients (132 women [75.4%]; mean [SD] age, 49.3 [12.5] years; mean [SD] BMI, 41.3 [9.1]) in the analysis at 3 months and 102 patients at 6 months. The mean (SD) weight loss after 3 months was 6.7 (4.4) kg, equivalent to a mean (SD) weight loss of 5.9% (3.7%) (*P* < .001), and the mean (SD) weight loss after 6 months was 12.3 (6.6) kg, equivalent to a mean (SD) weight loss of 10.9% (5.8%) (*P* < .001 from baseline). Of the 102 patients who were followed up at 6 months, 89 (87.3%) achieved weight loss of 5% or more, 56 (54.9%) achieved weight loss of 10% or more, 24 (23.5%) achieved weight loss of 15% or more, and 8 (7.8%) achieved weight loss of 20% or more. Patients with type 2 diabetes had a lower mean (SD) percentage weight loss at 3 and 6 months compared with those without type 2 diabetes: 3.9% (3.1%) vs 6.3% (3.7%) at 3 months (*P* = .001) and 7.2% (6.3%) vs 11.8% (5.3%) at 6 months (*P* = .005).

**Conclusions and Relevance:**

The results of this cohort study suggest that weekly 1.7-mg and 2.4-mg doses of semaglutide were associated with weight loss similar to that seen in randomized clinical trials. Studies with longer periods of follow-up are needed to evaluate prolonged weight loss outcomes.

## Key Points

**Question:**

Is treatment with semaglutide associated with weight loss outcomes similar to those seen in results of randomized clinical trials?

**Findings:**

In this cohort study of 175 patients with overweight or obesity, the total body weight loss percentages achieved were 5.9% at 3 months and 10.9% at 6 months.

**Meaning:**

Semaglutide treatment in a regular clinical setting was associated with weight loss similar to that seen in randomized clinical trials, which suggests its applicability for treating patients with overweight or obesity.

## Teaser

This cohort study assesses weight loss outcomes of semaglutide at doses used in randomized clinical trials for patients with overweight or obesity.

## Introduction

Obesity is a chronic, multifactorial, and relapsing disease^1^ with an increasing prevalence estimated to reach 49% by 2030.^2^ Its medical burden includes multiple comorbidities, such as type 2 diabetes, hypertension, dyslipidemia, stroke, coronary heart disease, and various cancers.^3^ A total cost of $1.71 trillion in the United States was attributed to chronic diseases associated with obesity.^4^ Considering the medical and economic costs associated with obesity, effective weight management is important to mitigate the associated morbidity and mortality.

Multiple weight loss interventions have been developed during the past decades. They include lifestyle and behavioral interventions (eg, diet and exercise), antiobesity medications (AOMs), endoscopic interventions, and surgical procedures.^5^ Antiobesity medications are an effective treatment for weight loss, aiming to improve quality of life and control weight-related comorbidities.^6,7,8^ However, different AOMs have shown a wide range of variability in percentage weight loss, ranging between 5% and 12% in randomized clinical trials (RCTs)^9^ and regular clinical settings.^10^ Currently, only 5 medications—orlistat, phentermine plus topiramate, naltrexone plus bupropion, liraglutide, and semaglutide—have been approved by the US Food and Drug Administration (FDA) for long-term use in individuals with a body mass index (BMI; calculated as weight in kilograms divided by height in meters squared) of 30 or more with no weight-related comorbidities or 27 or more with weight-related comorbidities.^11^

Semaglutide, a glucagon-like peptide-1 receptor agonist, is approved to treat type 2 diabetes,^12^ with subcutaneous injection doses of 0.25, 0.5, and 1 mg administered once weekly and oral doses of 3, 7, and 14 mg administered once daily.^13^ In June 2021, the FDA approved subcutaneous semaglutide for long-term weight management,^14^ with higher doses of 1.7 and 2.4 mg once weekly.^15^ The Semaglutide Treatment Effect in People With Obesity (STEP) trials have shown the efficacy of semaglutide for the treatment of obesity.^16^ In large RCTs, patients receiving semaglutide, 2.4 mg, lost a mean of 6% of their weight by week 12 and 12% of their weight by week 28.^16^ To our knowledge, no retrospective studies have been conducted to assess the effectiveness of semaglutide at the doses used to treat obesity (ie, 1.7 and 2.4 mg). In this study, we aim to assess weight loss outcomes associated with semaglutide treatment for patients with overweight or obesity and for patients with or without type 2 diabetes.

## Methods

### Study Design and Eligibility Criteria

We performed a retrospective review of the electronic medical records (EMRs) of patients in the Mayo Clinic Health System using semaglutide between January 1, 2021, and March 15, 2022. The Mayo Clinic institutional review board approved the study and waived the need for informed consent owing to its minimal-risk nature. We included patients who had at least a 3-month follow-up documented in the EMR with a BMI of 27 or more who were prescribed weekly semaglutide subcutaneous injections of 0.25, 0.5, 1, 1.7, and 2.4 mg with the primary goal of weight loss. We excluded patients with a history of bariatric procedures (ie, surgical or endoscopic), taking other FDA-approved AOMs, or with an active malignant neoplasm or pregnancy. Owing to high insurance denials and the national shortage of semaglutide, we subsequently excluded those patients from the analysis. This study followed the Strengthening the Reporting of Observational Studies in Epidemiology (STROBE) reporting guideline.

### Patient Selection

**Figure 1.**
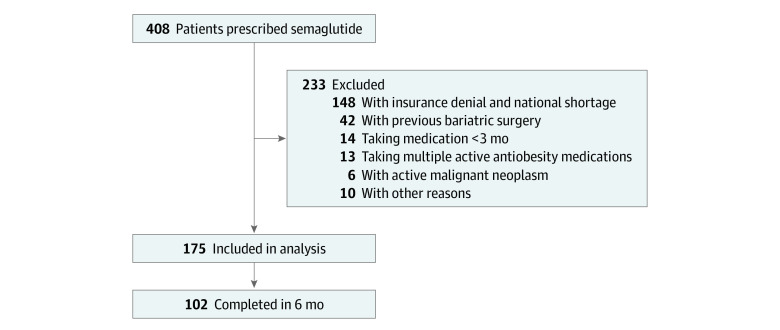
Flowchart of the Study

There were 408 prescriptions of subcutaneous injections of semaglutide between January 1, 2021, and March 15, 2022, at the Mayo Clinic Health System. We excluded 233 patients (insurance denial and national shortage of semaglutide, n = 148; previous bariatric surgery, n = 42; <3 months of semaglutide treatment, n = 14; multiple active AOMs, n = 13; active malignant neoplasm, n = 6; and other reasons, n = 10) (Figure 1). Detailed exclusion criteria are presented in eTable 1 in the Supplement.

### Data Collection

We abstracted demographic, anthropometric, and laboratory data within 30 days before or after 3 and 6 months. We collected patient weights in kilograms that were obtained either using a calibrated scale during office visits or reported by the patients during virtual visits and clinical communications with the physician. We also collected information on visits with dietitians and behavioral bariatric psychologists after the first day of starting semaglutide until the last day of follow-up or until the medication was discontinued. In addition, we collected information on any adverse effects experienced after the initiation of semaglutide treatment. During data abstraction, we confirmed the medication start date from physicians’ EMR notes or physician-patient communications because there might be a delay between the day of prescription and the start of medication (eg, insurance approval delay and drug availability).

### Study End Points

The primary end point was percentage weight loss at 3 and 6 months after semaglutide initiation, which was calculated using the following formula: Percentage Weight Loss = 100 × ([Weight at Baseline Visit − Weight at Follow-up Visit] / Weight at Baseline Visit). Our secondary end points included the percentage of patients who achieved a categorical weight loss of 5% or more, 10% or more, 15% or more, and 20% or more. We performed a post hoc analysis of patients with or without type 2 diabetes and of patients receiving different doses of semaglutide.

### Statistical Analysis

The baseline demographic and anthropometric data were normally distributed and are summarized as mean (SD) values. The primary end point was analyzed using the matched paired *t* test. For secondary end points, categorical data were analyzed using the Fisher exact test, and the 2-sample independent *t* test was used for continuous data. Statistical significance was set at 2-sided *P* < .05. We used JMP, version 16 (SAS Institute Inc) to perform the statistical analysis.

## Results

A total of 175 patients (132 women [75.4%]; 154 White patients [88.0%]; mean [SD] age, 49.3 [12.5] years; mean [SD] BMI, 41.3 [9.1]) of 408 patients with semaglutide prescriptions were included in the final cohort for analysis (Figure 1 and Table 1). Of the 175 patients, 102 had weight data at 6 months. Seventy-three patients did not have weight data at 6 months for several reasons: 61 (83.6%) started the medication less than 6 months prior, 7 (9.6%) had no recorded weights at 6 months when the data were extracted, 3 (4.1%) stopped the medication owing to adverse effects, and 2 (2.7%) stopped the medication owing to cost.

**Table 1.** Demographic and Clinical Characteristics

Characteristic	Patients (N = 175)
Demographic information	
Age, mean (SD), y	49.3 (12.5)
Sex, No. (%)	
Female	132 (75.4)
Male	43 (24.6)
Race, No. (%)	
Asian	9 (5.1)
Black	10 (5.7)
White	154 (88.0)
Not disclosed	2 (1.1)
Baseline clinical and laboratory information	
Weight, mean (SD), kg	118.1 (29.8)
BMI, mean (SD)	41.3 (9.1)
Overweight, No. (%)	5 (2.9)
Obesity, No. (%)^a^	
Class 1	41 (23.4)
Class 2	40 (22.9)
Class 3	89 (50.9)
Systolic blood pressure, mean, (SD), mm Hg	129 (16)
Diastolic blood pressure, mean (SD), mm Hg	78 (22)
Glucose, mean (SD), mg/dL (n = 121)	112 (38)
Hemoglobin A_1c_, mean (SD), % (n = 69)	5.9 (1.2)
Total cholesterol, mean (SD), mg/dL (n = 121)	183 (51)
Total triglycerides, mean (SD), mg/dL (n = 122)	144 (76)
LDL cholesterol, mean (SD), mg/dL (n = 119)	104 (40)
HDL cholesterol, mean (SD), mg/dL (n = 122)	50 (17)
Obesity comorbidities, No. (%)	
Dyslipidemia	80 (45.7)
Type 2 diabetes	28 (16.0)
Hypertension	86 (49.1)
GERD	56 (32.0)
Obstructive sleep apnea	63 (36.0)
NAFLD	67 (38.3)
Visit information	
Patient visits, No. (%)	
With dietitian	44 (25.1)
With psychologist	17 (9.7)
Patients with follow-up	
3 mo	175 (100)
6 mo	102 (58.3)

Abbreviations: BMI, body mass index (calculated as weight in kilograms divided by height in meters squared); GERD, gastroesophageal reflux disease; HDL, high-density lipoprotein; LDL, low-density lipoprotein; NAFLD, nonalcoholic fatty liver disease.

SI conversion factors: To convert glucose to millimoles per liter, multiply by 0.0555; hemoglobin A_1c_ to proportion of total hemoglobin, multiply by 0.01; total cholesterol, HDL cholesterol, and LDL cholesterol to millimoles per liter, multiply by 0.0259; and triglycerides to millimoles per liter, multiply by 0.0113.

^a^ Class 1 obesity: BMI of 30 to less than 35; class 2 obesity: BMI of 35 to less than 40; and class 3 obesity: BMI of 40 or higher.

### Baseline Characteristics

In our cohort of 175 patients, 89 patients (50.9%) had class 3 obesity (BMI, ≥40). Hypertension was the predominant comorbidity (86 [49.1%]), followed by dyslipidemia (80 [45.7%]) and nonalcoholic fatty liver disease (67 [38.3%]) (Table 1). The prevalence of type 2 diabetes was 16.0% (n = 18). Mean values of fasting glucose, hemoglobin A_1c_, total cholesterol, low-density lipoprotein cholesterol, high-density lipoprotein cholesterol, and triglycerides at baseline are presented in Table 1.

### Changes in Total Body Weight

**Figure 2.**
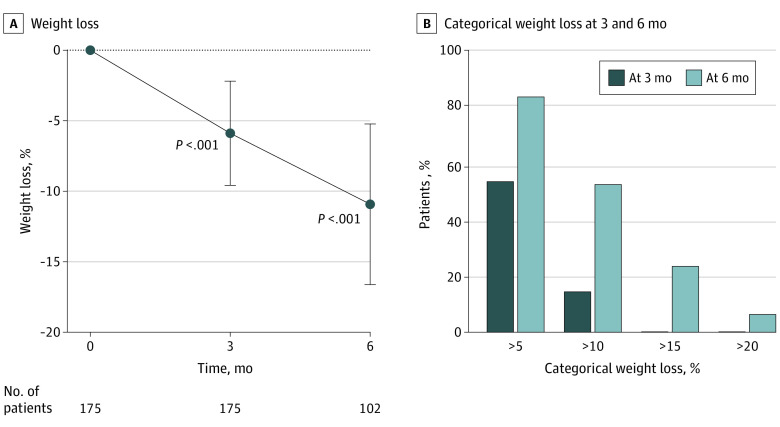
Percentage Weight Loss and Categorical Percentage Weight Loss at 3 and 6 Months Whiskers in panel A indicate SD.

At 3 months, 175 patients achieved a mean (SD) weight loss of 6.7 (4.4) kg, equivalent to a mean (SD) weight loss of 5.9% (3.7%) (*P* < .001 from baseline). At 6 months, 102 patients had a mean (SD) weight loss of 12.3 (6.6) kg, equivalent to a mean (SD) weight loss of 10.9% (5.8%) (*P* < .001 from baseline) (Figure 2). Visits with the dietitian or psychology teams were not associated with a greater percentage of weight loss (eTable 3 in the Supplement).

### Proportion of Patients Achieving 5% or More to 20% or More Weight Loss

In our cohort of 175 patients, 94 (53.7%) had weight loss of at least 5% and 26 (14.9%) had weight loss of 10% or more at 3 months. Of 102 patients who were followed up for 6 months, 89 (87.3%) achieved weight loss of 5% or more, 56 (54.9%) achieved weight loss of 10% or more, 24 (23.5%) achieved weight loss of 15% or more, and 8 (7.8%) achieved weight loss of 20% or more (Figure 2).

### Weight Loss by Diabetes Status

**Figure 3.**
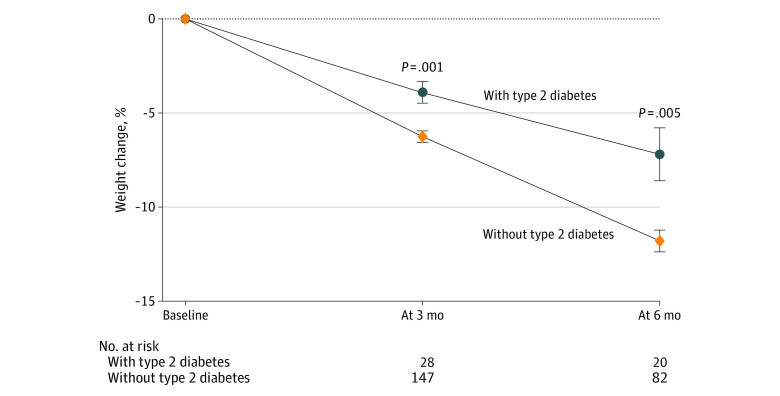
Percentage Weight Change at 3 and 6 Months for Patients With and Without Type 2 Diabetes Whiskers indicate SE.

Patients with type 2 diabetes had a lower mean (SD) percentage weight loss compared with those without type 2 diabetes at 3 months (3.9% [3.1%] vs 6.3% [3.7%]; *P* = .001) and at 6 months (7.2% [6.3%] vs 11.8% [5.3%]; *P* = .005) (Figure 3). Of the 28 patients with type 2 diabetes, 11 (39.3%) were using a combination of insulin with metformin, empagliflozin, and/or glipizide.

### Weight Loss by Obesity Class

Obesity classes are divided into class 1 (BMI, 30 to <35), class 2 (BMI, 35 to <40), and class 3 (BMI, ≥40).^17^ Patients with class 3 obesity had similar mean (SD) weight loss outcomes when compared with patients with overweight or classes 1 and 2 obesity at 3 months (7.1 [5.0] kg [n = 89] vs 6.3 [3.6] kg [n = 86]; *P* = .27), equivalent to mean (SD) weight loss of 5.3% (3.8%) vs 6.5% (3.6%) (*P* = .03) and at 6 months (−12.6 [7.9] kg [n = 51] vs −12.1 [5.0] kg [n = 51]; *P* = .70), equivalent to mean (SD) weight loss of 9.2% (5.9%) vs 12.6% (5.2%) (*P* = .002).

### Weight Loss With Different Semaglutide Doses

In our study, 77 patients (44.0%) received the highest current doses of subcutaneous semaglutide (1.7 and 2.4 mg), while 98 (56.0%) received lower doses (0.25, 0.5, and 1 mg). Detailed information of the maximum dose reached by our cohort of patients is presented in eTable 2 in the Supplement.

At 3 months, patients who received the highest doses achieved a mean (SD) weight loss of 6.9% (3.9%) (95% CI, −7.8% to −6.0% [n = 77]) compared with a mean (SD) weight loss of 5.1% (3.4%) (95% CI −5.8% to −4.4% [n = 98]) for patients receiving lower doses (*P* = .002). At 6 months, patients receiving the highest doses achieved a mean (SD) weight loss of 12.1% (5.9%) (95% CI, −13.6% to −10.6% [n = 60]) compared with a mean (SD) weight loss of 9.2% (5.2%) (95% CI, −10.9% to −7.6% [n = 42]) for patients receiving lower doses (*P* = .01) (eFigure 1 in the Supplement).

Our cohort experienced a wide range of weight loss responses to semaglutide at 3 and 6 months (eFigure 2 in the Supplement). Weight changes ranged between 3.6% and −14.3% at 3 months and between −0.6% and −29.1% at 6 months.

### Adverse Effect Profile

In our study, 85 patients (48.6%) reported experiencing adverse effects associated with semaglutide. Gastrointestinal symptoms were the most reported adverse effects. Nausea and vomiting were the most encountered adverse events (64 patients [36.6%]), followed by diarrhea (15 patients [8.6%]) and fatigue (11 patients [6.3%]). Table 2 presents a detailed report of adverse effects. Moreover, 5 patients (2.9%) had to stop semaglutide because of the intolerability of the adverse effects, while 15 (8.6%) had to either reduce the dose or remain on the same dose to avoid exacerbation of the adverse effects.

**Table 2.** Adverse Effects and Their Severity

Adverse effect	No. (%) (N = 175)
Any adverse effect	85 (48.6)
Nausea and vomiting	64 (36.6)
Diarrhea	15 (8.6)
Fatigue	11 (6.3)
Constipation	10 (5.7)
Abdominal pain	9 (5.1)
Headache	5 (2.9)
Acid reflux	4 (2.3)
Others^a^	8 (4.8)
Adverse effect severity	
None	90 (51.4)
Mild^b^	65 (37.1)
Moderate^b^	15 (8.6)
Severe^b^	5 (2.9)

^a^ Other adverse effects included dizziness, depression, bloating, dry mouth, and taste change.

^b^ Mild adverse effects did not affect dose escalation; moderate adverse effects prevented dose escalation; and severe adverse effects resulted in medication termination.

## Discussion

To our knowledge, this study is the first retrospective study to evaluate weight loss outcomes associated with treatment with subcutaneous semaglutide, including the highest doses approved for weight loss (>1.0 mg) for patients with overweight or obesity, with or without type 2 diabetes. In our cohort, patients lost approximately 6.7 kg at 3 months and 12.3 kg at 6 months, equivalent to 5.9% of weight lost at 3 months and 10.9% of weight lost at 6 months. Hence, this study may be a stepping stone to demonstrating the effectiveness of semaglutide for patients aiming to lose weight. These results may support the applicability of semaglutide in a less controlled environment, as previously proven in RCTs.^18,19,20^

### Comparison With RCTs

Several RCTs evaluated the weight loss outcomes of semaglutide. In one study including 1306 patients taking semaglutide, 2.4 mg, weight loss of approximately 6% was achieved by week 12 and 12% was achieved by week 28.^18^ Our results reflect similar weight loss outcomes within the same period, particularly for patients taking doses of 1.7 mg and 2.4 mg.

In the STEP 2 trial comparing 1 mg and 2.4 mg of semaglutide for patients with type 2 diabetes, similar weight loss trends compared with those in our study were seen. In 12 weeks, weight loss of approximately 4% was achieved among patients taking the 1-mg dose, and weight loss of approximately 5% was achieved among those taking the 2.4-mg dose.^21^ In addition, weight loss at 28 weeks approached approximately 7% for patients taking the 1-mg dose and 9.6% for those taking the 2.4-mg dose. Patients without type 2 diabetes achieved higher weight loss outcomes than those with type 2 diabetes, which is also shown in our study.^19^

In addition, considering the 16-week semaglutide titration schedule and having only 44.0% of the patients in our cohort reach maximal semaglutide doses (ie, 1.7 or 2.4 mg) compared with more than 94% of patients in RCTs,^18^ weight loss is expected to increase as more patients achieve the maximal doses of this medication.

### Comparison With Other AOMs

Considering the scarcity of AOMs, choosing the most suitable and individualized therapy is important.^6^ Retrospective studies comparing high doses of semaglutide (ie, 1.7 and 2.4 mg) with other AOMs are limited. A 2021 systematic literature review showed that 14% to 58.6% of patients achieved weight loss of 5% or more within 3 to 6 months when using orlistat, phentermine-topiramate, naltrexone-bupropion, phentermine, and liraglutide.^22^ However, in our study, 53.7% of patients at 3 months and 87.3% at 6 months had at least 5% weight loss. In a multicenter clinical experience assessing the outcome of FDA-approved AOMs (eg, phentermine-topiramate, liraglutide, orlistat, and naltrexone-bupropion), weight loss of 5.0% was achieved at 3 months compared with 5.9% in the present study, and weight loss of 6.8% was achieved at 6 months compared with 10.9% in the present study. In a study assessing the association of liraglutide, 3.0 mg, with weight loss, weight loss of 7.1% was achieved among patients followed up for 6 months or more, compared with weight loss of 10.9% in our study.^23^ In another study comparing orlistat (120 mg) with liraglutide (≤3.0 mg), both AOMs resulted in weight loss outcomes at 6 months or more of 3.3 kg for orlistat and 7.7 kg for liraglutide.^24^ A network meta-analysis comparing the weight loss outcomes of different glucagon-like peptide-1 receptor agonists demonstrated the following weight loss trends, from highest to lowest weight loss achieved: subcutaneous semaglutide, 2.4 mg weekly, −9.9 kg (95% CI, −13.2 to −6.6 kg); subcutaneous liraglutide, more than 1.8 mg daily, −4.5 kg (95% CI, −5.3 to −3.7 kg); subcutaneous semaglutide, less than 2.4 mg weekly, −4.3 kg (95% CI, −5.7 to −3.0 kg); oral semaglutide (3, 7, and 14 mg) daily, −2.7 kg (95% CI, −4.8 to −0.7 kg); subcutaneous liraglutide, 1.8 mg or less daily, −2.7 kg (95% CI, −3.4 to −2.1 kg); subcutaneous extended-release exenatide (0.8 and 2.0 mg) weekly, −2.2 kg (95% CI, −4.3 to −0.1 kg); subcutaneous immediate-release exenatide (10, 20, 40, and 60 μg/d) twice daily, −1.8 kg (95% CI, −2.4 to −1.2 kg); subcutaneous dulaglutide, 1.5 mg or more weekly, −1.4 kg (95% CI, −2.1 to −0.7 kg); and subcutaneous lixisenatide (10, 15, and 20 μg) daily, −0.6 kg (95% CI, −1.2 to −0.02 kg).^25^ Moreover, in a retrospective trial for patients receiving phentermine, weight loss of 4.9% was achieved in 12 weeks^26^ compared with weight loss of 5.9% with semaglutide in the same period. In another observational study, 233 patients receiving phentermine-topiramate had a weight loss of 4.1%, while 304 patients receiving phentermine achieved a weight loss of 3.6% in approximately 24 weeks of follow-up.^27^ In an RCT studying the weight loss outcomes of 32 mg of sustained-release naltrexone and 360 mg of sustained release bupropion in combination with a comprehensive lifestyle intervention program, patients had a mean weight loss of 9.5% in 26 weeks.^28^ Hence, the weight loss outcomes associated with semaglutide in our study seem to be superior to the outcomes with other AOMs, although head-to-head trials would be needed to compare outcomes in a controlled setting.

In our study, patients with type 2 diabetes lost less weight compared with those without type 2 diabetes. This difference was also seen in previous studies with semaglutide^19,21^ and in other studies in which patients with type 2 diabetes had a weight loss inferior to that of matched patients without type 2 diabetes.^29,30^ A possible explanation could be a greater decrease in energy expenditure among patients with type 2 diabetes compared with those without type 2 diabetes.^29,31^ In addition, weight loss results in an improvement of glucose control, leading to a decrease in glycosuria. Consequently, this creates a positive calorie balance, making it more difficult to lose weight.^29^ Another explanation includes other antidiabetic medications that are associated with weight gain (eg, insulin and glipizide).^32,33^

In our study, adverse effects were reported in approximately 50% of our cohort during follow-up. In other RCTs following up with patients treated with semaglutide, 2.4 mg, adverse effects were reported in 90% of patients over 68 weeks.^18^ Some possible explanations for the lower reported adverse effects in our study include patients not reaching the maximum dose of semaglutide (ie, 1.7 and 2.4 mg) and the shorter duration of follow-up (24 vs 68 weeks). Similar to our reported results, gastrointestinal symptoms, particularly nausea, are the most commonly reported adverse effects with semaglutide.^18^ We also report a significant number of patients with fatigue, which was not reported in previous studies.^18^

Moreover, we reported few moderate and severe adverse events (eg, nausea and constipation) that resulted in continuing to receive the same dose of medication or even discontinuing semaglutide. However, patients who were lost to follow-up may have experienced adverse effects and discontinued the medication without reporting to the clinician. This possibility might be one explanation for our relatively lower rate of medication discontinuation compared with the 7% reported in the STEP 1 trial.^18^ As described in previous studies, no unexpected safety issues were recorded for our cohort of patients.^34^

### Strengths and Limitations

This study has some strengths, including an adequate sample size at 3 and 6 months of follow-up. In addition, the weight loss data of semaglutide use might be more representative of day-to-day clinical practice, including a more heterogenous patient population, compared with RCTs.

This study also has some limitations. Considering the observational nature of this study, we could not compare weight loss outcomes between patients receiving semaglutide and controls. The weight loss achieved may be associated with other interventions (eg, lifestyle interventions and diet) that are provided by the weight management clinic; however, we found that visits with a dietitian and a psychology group were not associated with greater weight loss. Another serious limitation is the exclusion of patients who did not reach 3 months of follow-up, which might have resulted in an overestimation of the association of semaglutide with body weight. In addition, we had mostly White female patients, which limits the generalizability of the study. Also, there is a possibility of recall bias because the dates of medication initiation and termination were reported by patients during patients’ visits or communications with their physicians and therefore may not be exact. Moreover, some of the abstracted weights were self-reported, which might be not as accurate as clinic measurements (noncalibrated vs calibrated scales). Finally, using the EMR database might have increased susceptibility to coding errors and missing data during the data extraction phase. For example, any information (eg, on adverse effects) that was not reported by the patient or entered into the EMR portal by the health care professional could potentially be missed. In addition, some of the missing data may be associated with drug discontinuation for various reasons (eg, adverse effects, cost of the medication, lack of effectiveness, or patient’s decision) that are hard to identify based on the retrospective nature of this study.

## Conclusions

The findings of this cohort study suggest that semaglutide is clinically effective for weight loss at 3 and 6 months for people with overweight or obesity. Although our study lacked the stringent and closely controlled nature of RCTs, we report similar weight loss results within the same time period as in RCTs. Studies with greater sample sizes and longer periods of follow-up are further needed to support the effectiveness of semaglutide.

## Notes

Supplement. **eTable 1.** Detailed Exclusion Criteria **eTable 2.** Maximum Dose of Semaglutide Reached **eTable 3.** Percentage Weight Change of Patients Taking Semaglutide With/Without Dietitian or Psychologist Visits at 3 and 6 Months **eFigure 1.** Total Body Weight Loss Percentage (TBWL%) at 3 and 6 Months of Patients Taking Low and High Doses of Subcutaneous Semaglutide **eFigure 2.** Percentage Weight Change Heterogeneity in Patients Taking Semaglutide at 3 and 6 Months
