# Supplementary material for: Weight Loss Outcomes Associated With Semaglutide Treatment for Patients With Overweight or Obesity
Source: JAMA Netw Open. 2022 Sep 19;5(9):e2231982. doi: 10.1001/jamanetworkopen.2022.31982 (PMC9486455; doi:10.1001/jamanetworkopen.2022.31982)
Supplement: Supplement. — eTable 1. Detailed Exclusion Criteria eTable 2. Maximum Dose of Semaglutide Reached eTable 3. Percentage Weight Change of Patients Taking Semaglutide With/Without Dietitian or Psychologist Visits at 3 and 6 Months eFigure 1. Total Body Weight Loss Percentage (TBWL%) at 3 and 6 Months of Patients Taking Low and High Doses of Subcutaneous Semaglutide eFigure 2. Percentage Weight Change Heterogeneity in Patients Taking Semaglutide at 3 and 6 Months [file jamanetwopen-e2231982-s001.pdf]

Supplementary Online Content

Ghusn W, De la Rosa A, Sacoto D, et al. Weight loss outcomes associated with semaglutide treatment for patients with overweight or obesity. *JAMA Netw Open.* 2022;5(9):e2231982. doi:10.1001/jamanetworkopen.2022.31982

eTable 1. Detailed Exclusion Criteria

eTable 2. Maximum Dose of Semaglutide Reached

eTable 3. Percentage Weight Change of Patients Taking Semaglutide With/Without Dietitian or Psychologist Visits at 3 and 6 Months

eFigure 1. Total Body Weight Loss Percentage (TBWL%) at 3 and 6 Months of Patients Taking Low and High Doses of Subcutaneous Semaglutide

eFigure 2. Percentage Weight Change Heterogeneity in Patients Taking Semaglutide at 3 and 6 Months

This supplementary material has been provided by the authors to give readers additional information about their work.

© 2022 Ghusn W et al. *JAMA Network Open.*

eTable 1. Detailed Exclusion Criteria

Reasons for exclusion	Number of patients
Insurance denial and national shortage, (%)	148 (64)
Previous bariatric procedure, (%)	42 (18)
Less than 3 months on semaglutide, (%)	14 (6)
Multiple active anti-obesity medication, (%)	13 (6)
Active malignancy, (%)	6 (3)
No baseline weight, (%)	4 (2)
Medical disease affecting weight, (%)	3 (1)
Unknown semaglutide start date, (%)	3 (1)
Total, (%)	233 (100)

© 2022 Ghusn W et al. *JAMA Network Open.*

eTable 2. Maximum Dose of Semaglutide Reached

Maximum dose reached	Number of patients, (%)
0.25 mg	6 (3)
0.5 mg	36 (21)
1 mg	56 (32)
1.7 mg	29 (17)
2.4 mg	48 (27)
Total	175 (100)

© 2022 Ghusn W et al. *JAMA Network Open.*

eTable 3. Percentage Weight Change of Patients Taking Semaglutide With/Without Dietitian or Psychologist Visits at 3 and 6 Months

	**Dietician visits**			**Psychologist visits**		
	Yes	No	p-value	Yes	No	p-value
**Three Months (% weight loss)**	-5.9	-5.9	0.98	-5.7	-5.9	0.86
**Six Months (% weight loss)**	-9.8	-11.4	0.23	-10.3	-11	0.68

© 2022 Ghusn W et al. *JAMA Network Open.*

eFigure 1. Total Body Weight Loss Percentage (TBWL%) at 3 and 6 Months of Patients Taking Low and High Doses of Subcutaneous Semaglutide

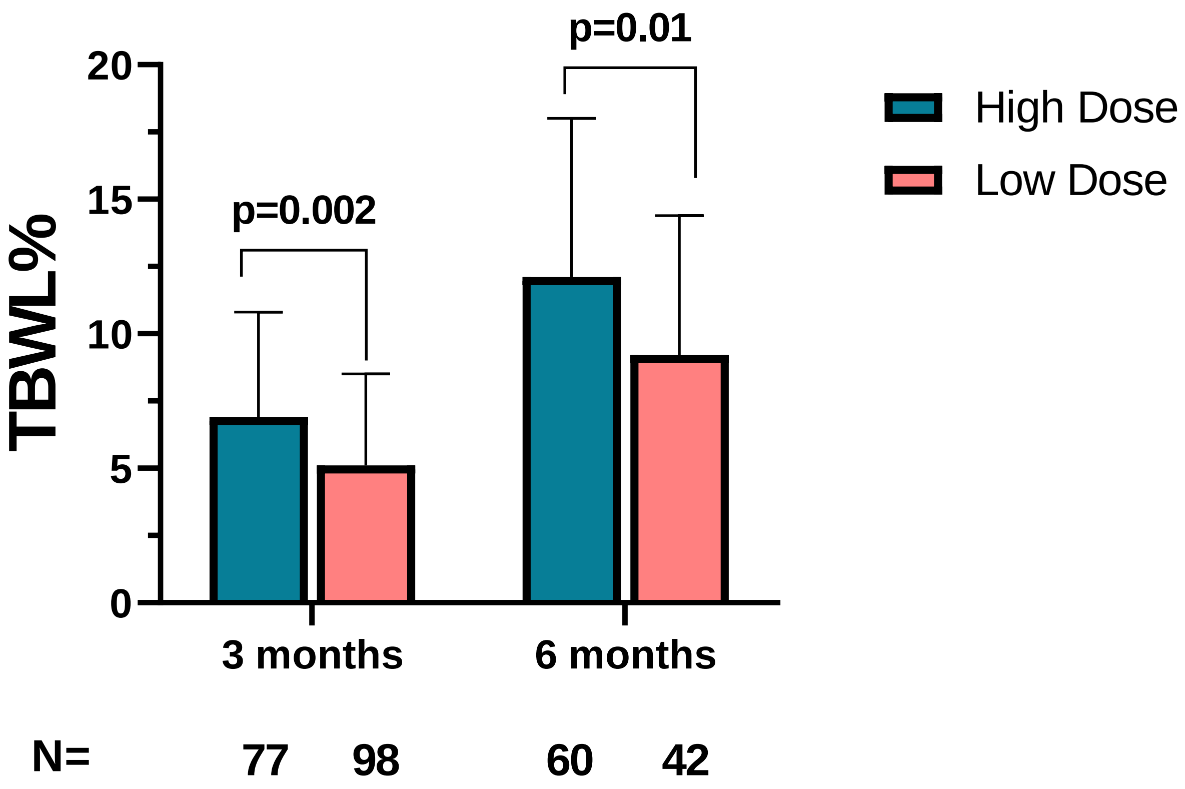

© 2022 Ghusn W et al. *JAMA Network Open.*

eFigure 2. Percentage Weight Change Heterogeneity in Patients Taking Semaglutide at 3 and 6 Months

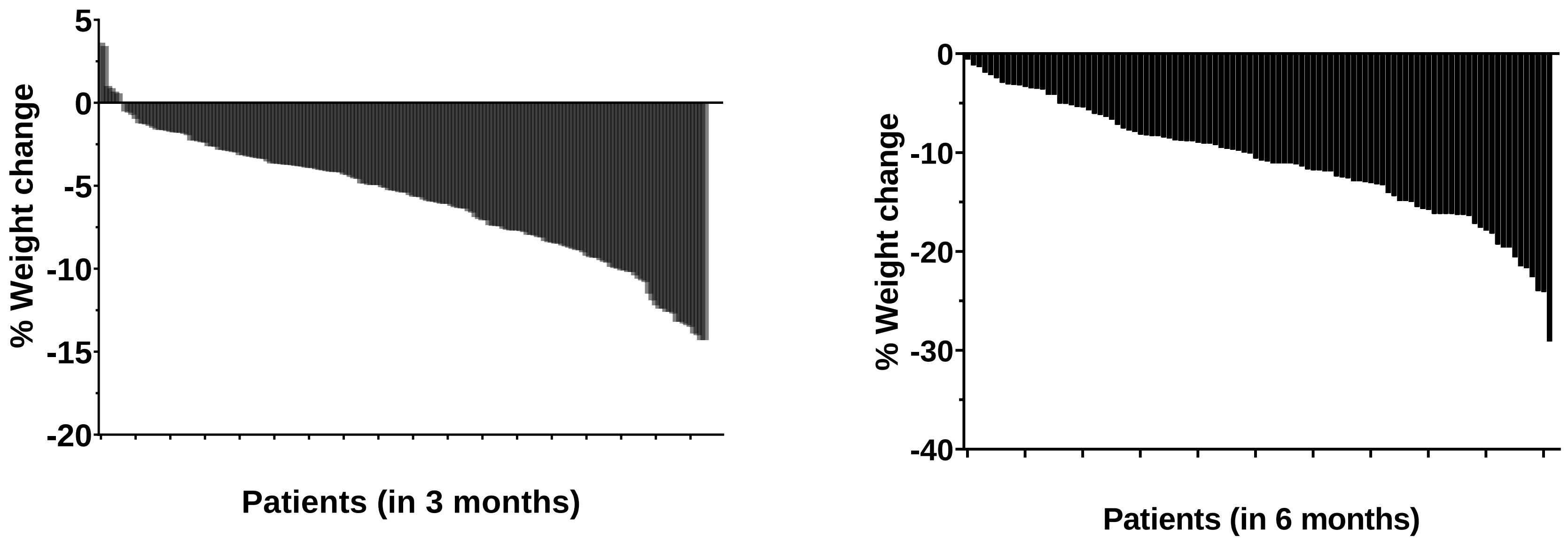

© 2022 Ghusn W et al. *JAMA Network Open.*
